# Long-Term Profiles of Bullying Victims and Aggressors: A Retrospective Study

**DOI:** 10.3389/fpsyg.2021.631276

**Published:** 2021-06-29

**Authors:** Mario Valera-Pozo, Albert Flexas, Mateu Servera, Eva Aguilar-Mediavilla, Daniel Adrover-Roig

**Affiliations:** ^1^Department of Applied Pedagogy and Educational Psychology, Institute of Research and Innovation in Education (IRIE), University of Balearic Islands, Palma, Spain; ^2^Department of Psychology, University Institute of Research in Health Sciences (IUNICS) and Institute of Sanitary Research of Balearic Islands (IDISBA), University of Balearic Islands, Palma, Spain

**Keywords:** aggression, victimization, scholar problems, emotion regulation, retrospective study

## Abstract

Bullying is a widespread and worrying phenomenon, related to many different personal, behavioral, and social variables which can modulate it and its outcomes, also in the long term. These relationships are usually studied in children and adolescents, but less often in adults who have suffered or perpetrated bullying in the past. The present work explored the long-term characteristics of bullying victims and aggressors using a retrospective design. A sample of 138 adults of different ages completed an on-line protocol that included measures of bullying and victimization, substance use, sensitivity to reward and punishment, social skills, antisocial behavior, emotional regulation strategies, depression, anxiety, stress, self-esteem, and risk of suicide. The sample was divided into three groups (victims, aggressors, and controls) based on their responses to bullying-related questions. A set of Multiple Analyses of Variance with group as a fixed factor was carried out for each dependent variable. Victims and aggressors did not significantly differ in their self-reported substance consumption. Victims showed higher global depression, anxiety and stress in the past than aggressors (*M* = 34.66, *SD* = 11.74; aggressors: *M* = 19.70, *SD* = 16.53), higher emotional lack of control (*M* = 23.97, *SD* = 10.62; controls: *M* = 17.11, *SD* = 7.95) and rejection (*M* = 21.72, *SD* = 7.24; controls: *M* = 16.33, *SD* = 5.67), lower self-esteem (*M* = 27.72, *SD* = 6.70; controls: *M* = 31.60, *SD* = 6.60), and a larger frequency of suicidal thoughts (in the past) than controls. Aggressors showed higher sensitivity to reward (*M* = 12.03, *SD* = 3.66; controls: *M* = 8.42, *SD* = 3.92), larger communicational and relational skills (*M* = 22.10, *SD* = 7.20; controls: *M* = 17.96, *SD* = 7.16), and lower emotional sensitivity (*M* = 14.80, *SD* = 4.10; controls: *M* = 16.76, *SD* = 2.21). Accordingly, the logistic regression analysis identified sensitivity to reward and low psychological adjustment as the main predictors of the aggressor and victim profiles, respectively. The present results are discussed considering the extant literature on bullying and may help to improve prevention programs for this relevant social scourge.

## Introduction

Norwegian professor Dan Olweus defined bullying in 1973 as an intentional and repeated behavior carried out by some pupils in order to cause harm or disturb (in different ways, including verbal, physical, psychological, and social domains) a peer with whom an asymmetric relationship is established (Olweus, 1998). According to Craig et al. (2009), the prevalence of bullying is up to 45% in some countries, and both victims and aggressors are at risk of long-term psychosocial and developmental consequences (for a review, see Zych et al., 2015). In Spain, the most recent report (Save the Children, 2016) indicates that 9.3% of students between 12 and 16 years of age consider themselves victims of bullying, while 5.4% consider themselves aggressors. However, long-term outcomes of this phenomenon have been little explored in our country.

Although bullying is often studied in childhood and adolescence, its psychosocial effects can carry on even in adulthood. As bullying research grows in interest, different studies have attended to its developmental pathways, seeking developmental causes or bullying trajectories in a longitudinal way. Shakoor (2012) offers a developmental view in which bullying involvement is mediated by a set of different aspects such as altered theory of mind skills (which can be related to attribution biases), antisocial behaviors, adjustment and emotional problems, and substance use.

Other authors (Pepler et al., 2008a,b; Swearer and Hymel, 2015) have depicted bullying from a developmental-systemic perspective. This perspective describes bullying as an addition of the factors of three agents: children themselves, their families and their relationships with peers. While victims are characterized by higher levels of depression and lower self-esteem, aggressors present more deviant behaviors. Pepler et al. (2008a) state that, despite the different profiles of aggression, bullying follows a certain pathway: a combination of high emotional sensitivity and unemotional traits (mainly, moral disengagement) with low social cognition, which may lead aggressors to their behavior. In the case of moderate and high trajectories of bullying, this schema evolves from elementary to high school: as social insight increases, aggressors might acquire more skills to read other vulnerabilities and manipulate them, using power and aggression to resolve relational problems and obtain social acceptance. In summary, when they grow, they use aggression to get what they want, in a perverse but adaptive way (Rodkin et al., 2015). In the long-term, these pathways lead not only to more psychosocial problems for both victims and aggressors, but also to more conflicts (Pepler et al., 2008b).

In this vein, Swearer and Hymel (2015) describe bullying as a stressful event for those who get involved in it, with a large list of psychosocial problems as an outcome: depression and anxiety, low self-esteem, suicidal ideation and substance abuse. Moreover, they identify bullying as a complex and volatile phenomenon through time. In this regard, bullying consequences can be developmentally explained with a model of diathesis-stress. In this model, several personal characteristics, together with this stressful problem, can lead to different psychopathologies and problems in social relations in the short and in the long term.

As mentioned, there are several variables that can influence the involvement in bullying, with a considerable amount of individual ones. Regarding the long-term perspective, substance use (Pérez Fuentes and Gázquez Linares, 2010) might be of crucial relevance. Consumption of alcohol and cannabis occurs for both victims and aggressors in adolescence (Antoniadou et al., 2016; Lee et al., 2018), a period in which personality is in development. In this regard, two crucial systems related with personality have been found to be also associated with aggression: sensitivity to reward and punishment avoidance (Šarić Drnas, 2020). Aggressors seem to be more sensitive to reward (Flouri and Papachristou, 2019) than adolescents not involved in bullying. On the contrary, sensitivity to punishment has been less explored in victimization. However, since victims show a more intensive hostile attribution bias and higher social anxiety (Hoglund and Leadbeater, 2007; Perren et al., 2013; Swearer and Hymel, 2015) it is plausible that they might experience a heightened sensitivity not only to potential aversive social situations, but also to punishment and to aversive stimuli in broader terms.

Other variables that also modulate and could derive from bullying refer to social and emotional aspects. Both victims and aggressors can present worse social and communicative skills and poorer social functioning (Sandoval et al., 2015; Kljakovic and Hunt, 2016). In the case of victims, this deficit is marked by the possible aversion to social situations and the presence of less adaptive strategies for conflict resolution (Nacimiento Rodríguez and Mora-Merchán, 2015; Garaigordobil, 2017) whereas in aggressors it has been related to antisocial behavior and callous/unemotional traits (Antoniadou et al., 2016; Garaigordobil, 2017). Besides these social functioning issues, the bully-victim interaction could be affected by poor emotional regulation strategies in front of conflicts, and certain patterns of emotional regulation may act as risk factor for chronic victimization (Mahady Wilton et al., 2000).

Other studies have also found that victims of bullying generally present higher anxiety and depression levels and show lower psychological adjustment compared to the ordinary population of adults (Forster et al., 2013; Stuart and Jose, 2014; Sandoval et al., 2015). They also tend to show low self-esteem (for a review, see Tsaousis, 2016) and even a higher frequency of suicidal thoughts and suicidal attempts (Shin Kim and Leventhal, 2008; Alavi et al., 2015).

Altogether, the extant literature suggests that bullying is related with several psychological implications and individual factors (besides familiar and relational aspects) that could extend over years. For this reason, we aimed to explore this issue through retrospective reports, which might allow a better understanding of the long-term relations of bullying and its patterns throughout the lifespan. Although retrospective studies are not exempt from risk of bias, confounding memories or forgetfulness (Hardt and Rutter, 2004), this method has demonstrated to be an adequate candidate to explore prior experiences of bullying or aversive events in adults and their long-term consequences (Rivers, 2001; Green et al., 2018). It has been also used in several fields of research focusing on different forms of maltreatment (Anda et al., 2006; Corso et al., 2008).

The main goal of this study was (G1) to analyze the potential long-term outcomes of having been a victim of bullying in the past and/or to have been an aggressor, when compared to a control group. We also aimed (G2) to identify the best predictors of being classified as a victim or as an aggressor by means of a logistic regression. We expected that, with respect to control group, both victims and aggressors would show currently more frequent use of alcohol and cannabis (H1) and poorer social skills (H2). We also expected that victims would show more sensitivity to punishment (H3), while aggressors would show higher sensitivity to reward (H4). Regarding victims, they would show worse emotional regulation strategies (H5) and higher depression, anxiety and stress levels (H6), a lower self-esteem (H7), and a larger frequency of suicidal thoughts, at least in the past (H8). In the case of aggressors, they were expected to present lower emotional sensitivity scores (H9).

## Materials and Methods

### Participants

A sample of 138 young and adult participants from the Balearic Islands (Spain) took part in the present study by means of an on-line protocol, which was distributed using several social and academic networks. Participants were aged between 17 and 35 years and there were 113 females (81.88% of the sample). Out of the sample, two participants were at high school (1.45%), 103 (74.64%) were coursing degree studies, and 33 (23.91%) had accomplished postgraduate studies. Data on the ethnicity of participants were also collected because bullying could affect differentially ethnic minorities (for a review, see Xu et al., 2020).

We created three groups of participants with the purpose to respond to the departing hypotheses: a group of former victims of bullying, a group of former aggressors, and a control group of non-victims who were non-aggressors. After providing the definition of bullying, the group of victims was configured based on the response to a single question: “*Have you ever suffered bullying?*” There were four possible answers: (1) Yes, continuously during one scholar year; (2) Yes, occasionally during one scholar year, (3) No, but I have witnessed it, and (4) No, and I have not witnessed it. Victims were considered as participants who answered affirmatively to the first option, and we excluded participants who also were considered aggressors from this group (see below). Twenty-nine participants (21.01% of the sample) stated to have suffered continuous bullying during at least one scholar year and not having perpetrated it.

For the group of aggressors, participants were classified according to the question “*Have you ever perpetrated bullying?*” There were three possible answers: (1) Yes, continuously during one scholar year; (2) Yes, occasionally during one scholar year, (3) No. Aggressors were considered as participants who answered affirmatively to the first or second options. The group of aggressors was composed of 30 participants (21.74%), which included “pure” aggressors (*n* = 8; 5.8%) and aggressors who also scored in victimization (*n* = 22, 15.94% of the sample). In this way, six participants in the aggressor group (20%) stated to be also occasional victims, 16 continuous victims (53.3%) and eight (26.6%) did not score on victimization. In this regard, it is worth noting that aggressors tend to score high in victimization, as stated in previous reports (Salmivalli and Nieminen, 2002).

Finally, the control group included participants (*n* = 45, 32.61% of the sample) who answered negatively to both questions: “*Have you ever suffered bullying?*” (answers 3 or 4), and “*Have you ever perpetrated bullying?*” (answer 3), and therefore were non-victims and non-aggressors.

The remaining 34 persons who answered the on-line protocol were not included in the present study because they stated to have suffered occasional bullying behaviors. We could not consider them as victims, according to its definition (Olweus, 1998), and we think that they could not be considered as part of the control group either, because they showed hints of victimization.

In sum, we obtained a group of “pure” victims who stated to have suffered bullying during at least one scholar year and did not report to have perpetrated bullying; a group of aggressors who stated to have perpetrated bullying either continually or occasionally during one scholar year, who also could score in victimization; and a control group that neither had been an aggressor nor a victim of bullying (see Table 1 for sample demographics).

**Table 1:** Total and percentage of victims, aggressors, and control participants according to age, degree, gender, and ethnicity.

	**Groups**			**Total**	
	**Victim**	**Aggressor**	**Control**	
	**Freq (%)**	**Freq (%)**	**Freq (%)**	**Freq (%)**	***Fisher Exact Test* (*p*)**
**Age groups**					
17–20	12 (31.6)	10 (26.3)	16 (42.1)	38 (36.5)	16.19 (0.011)
21–23	3 (14.3)	8 (38.1)	10 (47.6)	21 (20.2)	
24–25	8 (42.1)	0 (0)	11 (57.9)	19 (18.3)	
25–35	6 (23.1)	12 (46.1)	8 (30.8)	26 (25)	
**Degree^a^**					
Humanities	8 (50)	5 (31.2)	3 (18.8)	16 (16.3)	20.69 (0.001)
Science & engineering	4 (40)	4 (40)	2 (20)	10 (10.2)	
Health sciences	9 (25.7)	3 (8.6)	23 (65.7)	35 (35.7)	
Social sciences	6 (16.2)	15 (40.5)	16 (43.2)	37 (37.8)	
**Gender**					
Male	8 (34.8)	9 (39.1)	6 (26.1)	23 (22.1)	3.76 (0.147)
Female	21 (25.9)	21 (25.9)	39 (48.2)	81 (77.9)	
**Ethnicity**					
Caucasian	22 (27.1)	25 (30.9)	34 (42)	81 (77.9)	0.74 (0.725)
Other	7 (30.4)	5 (21.7)	11 (47.8)	23 (22.1)	

*p represents the significance level. Victims affirmed to have suffered continued bullying at least during one scholar year in the past, whereas aggressors stated to have perpetrated it either occasionally or continuously during a scholar year. Freq, frequency*.

a *There are 6 missing responses*.

### Materials

We administered a set of questionnaires compiled in the form of an on-line protocol, which also included several questions addressed to obtain sociodemographic data, such as age, education level achieved, specific University degree coursed (if applicable), ethnicity and gender. Participants did not provide their names or surnames in order to ensure their anonymity. The questionnaires used for data collection contained measures of alcohol and cannabis consumption, social skills, sensitivity to reward and to punishment, emotional regulation, depression, anxiety, stress, self-esteem, emotional sensitivity and risk of suicide. The average time to respond to the online protocol was 20 min.

To assess substances consumption, we used two different tests: the *Alcohol Use Disorders Identification Test* (AUDIT; Babor et al., 2001) and the *Cannabis Use Disorders Identification Test-Revised* (CUDIT-R; Adamson et al., 2010). As there were not Spanish adaptations available at the time of testing, we decided to follow a back-translation process for both questionnaires. Thus, we made an initial Spanish translation based on the original versions, which were afterwards back-translated by a professional translator. We compared the original and the new English back-translated version, and both were identical. After that process, we administered the versions adapted to Spanish.

The AUDIT (Babor et al., 2001) is a self-reported questionnaire which measures alcohol consumption in the last 6 months. It is composed of ten Likert-like items with five answer options each (“never,” “once a month or less,” “2–4 times a month,” “2–3 times per week,” and “4 or more times per week”; e.g., “How often do you have a drink containing alcohol?”). The addition of the 10 items reports a measure of the frequency of alcohol consumption. Higher scores in this variable indicate larger alcohol consumption. Its internal consistency reliability is α = 0.83 for the original questionnaire (Babor et al., 2001) and α = 0.77 for the current study.

The CUDIT-R (Adamson et al., 2010) is a self-reported test which measures cannabis consumption in the last 6 months. It is composed of eight Likert-like items of five answer options each (“never,” “once a month or less,” “2–4 times a month,” “2-3 times per week,” and “4 or more times per week”; e.g., “How often do you use cannabis?”). The addition of the eight items reports a measure of the frequency of cannabis consumption. Higher scores in this variable indicate larger cannabis consumption. Its internal consistency reliability is α = 0.87 for the original questionnaire (Adamson et al., 2010) and α = 0.76 for the current study.

The *Scale for the evaluation of Social Skills/Escala de Habilidades Sociales* (SESS/EHS; Oliva et al., 2011) was used to measure social skills. The SESS/EHS is a scale composed of 12 Likert-like items with seven answer options (from “totally false” to “totally true”). This scale is composed of three dimensions: communicative-relational skills (e.g., “It is hard to me to begin a conversation with a stranger”), assertiveness (e.g., “If I feel that someone is upset with me, I ask him/her why”) and conflict resolution abilities (e.g., “I usually mediate the arguments between my mates”). The addition of the correspondent items for each dimension offers the three different types of social skills abovementioned. Higher scores in any of the scores indicate better social skills in that field. For the original scale (Oliva et al., 2011), the internal consistency reliability is up to α = 0.80; meanwhile, it is up to α = 0.83 for the current study.

The *Sensitivity to punishment and sensitivity to reward/Sensibilidad al Castigo, Sensibilidad a la Recompensa* (SPSR/SCSR; Torrubia et al., 2001) was used to measure the sensitivity to reward and to punishment. The SPSR offers a measure for punishment sensitivity and another for reward sensitivity, providing an approximation of which of these scales influences more the daily life of participants. The SPSR is a questionnaire composed of 48 items (24 for punishment sensitivity—e.g., “Do you prefer not asking for something if you are sure that people will not give it to you?”— and 24 for reward sensitivity—e.g., “Do you often do things to be praised?”), which are responded by means of a dichotomic true/false answer. The addition of the affirmative responses for each variable compounds the two total scores. Higher scores in those measures indicate larger sensitivity to reward and punishment, respectively. Its internal consistency reliability is α = 0.83 for punishment sensitivity and α = 0.78 for reward sensitivity for the original questionnaire (Torrubia et al., 2001), meanwhile for the current study is α = 0.87 for punishment sensitivity and α = 0.74 for reward sensitivity.

The Spanish version of *Difficulties in Emotion Regulation Scale* (DERS; Gratz and Roemer, 2004; Spanish adaptation by Hervás and Jódar, 2008) was used to assess emotional regulation. The DERS is a scale composed of 28 items in its Spanish adaptation. The items are answered with a Likert response (five options, from “almost never” to “almost always”). The sum of the items of this scale gives a global score of emotional maladjustment and a broken down measure of five dimensions: emotional lack of control (measuring emotional impulsivity and deficits for its control; e.g., “When I feel bad, I lose control”), emotional rejection (non-acceptance of emotions; e.g., “When I feel bad, I am embarrassed for feeling that way”), life interference (difficulties in goal-directed behavior; e.g., “When I feel bad, I have difficulties to concentrate”), lack of emotional attention (lack of emotional conscience; e.g., “I am attentive to my feelings”) and emotional confusion (lack of emotional clarity; e.g., “I am confused about my feelings”). Higher scores in the global measure reflect larger difficulties in emotional regulation, meanwhile higher scores in the dimensions show larger difficulties in these specific fields. The internal consistency reliability for the Spanish adaptation (Hervás and Jódar, 2008) is α = 0.93 for the global score, fluctuating between α = 0.73 and α = 0.91 depending on the dimensions. For the current study, the internal consistency reliability is α = 0.95 for the global score, and between α = 0.83 and α = 0.95 depending on the dimensions.

We also administered the Spanish version of the *Depression Anxiety Stress Scales-21* (Lovibond and Lovibond, 1995; DASS-21; Spanish adaptation by Arturo et al., 2005) to measure these psychological problems in the past, when the bullying situation occurred (using a retrospective version of it), and at the moment of testing. The DASS-21 is an abbreviated scale composed of 21 items which are answered with a Likert-type response including four answer options (from “not applicable to me” to “very applicable to me, most of the time”). This test measures the levels of depression (e.g., “I cannot feel any positive emotion”), anxiety (e.g., “I feel about to panic”) and stress (e.g., “It is very difficult to me to discharge stress”) suffered by the participant in the last 7 days, adding the responses of the seven items of each variable to calculate them. The total score provides a proxy of current psychological problems, where higher values indicate larger depression, anxiety, and stress. Its internal consistency reliability for the Spanish version of the questionnaire (Arturo et al., 2005) is between α = 0.70 and α = 0.84, depending on the dimension. For the current study, it fluctuates between α = 0.76 and α = 0.85, with a global score reliability of α = 0.91.

The Spanish version of the *Rosenberg Self-Steem Scale* (Rosenberg, 1989; RSES; Spanish adaptation by Martín-Albo et al., 2007) was applied to measure self-esteem. The RSES is a brief scale composed of 10 items. These items are Likert-type with four answer options (from “totally disagree” to “totally agree”; e.g., “I have a positive attitude toward myself”) and its sum provides a global score of the sense of satisfaction that a person has with himself/herself. Higher values in the total score are indicative of a larger and better self-esteem. Its internal consistency reliability is α = 0.88 for the Spanish adaptation (Martín-Albo et al., 2007) and α = 0.90 for the current study.

The *Limited Prosocial Emotions* (LPE-Cut; American Psychiatric Association, 2013) was used to measure emotional insensitivity, a nuclear trait of persons who present antisocial behavior. The LPE specifier acts here as a brief questionnaire composed of a global score obtained from adding the four Likert-like items with six response options (from “almost never” to “almost always”; e.g. “You feel guilty when you make a bad behavior”). Lower scores indicate higher emotional insensitivity. Its internal consistency reliability for the current study is α = 0.61.

Finally, in order to measure the risk of suicide, we adopted a series of *ad-hoc* questions, which are based on the risk of suicide items from the *Mini International Neuropsychiatric Interview* (Sheehan et al., 1992; MINI; Spanish adaptation by Ferrando et al., 1999). Participants answered three yes/no response questions, which referred to both the last month and related to the past, which were: “*Have you felt so bad that you wanted to be dead?,” “Have you ever tried to hurt yourself?,”* and “*Have you ever tried to take your own life?”* According to the MINI, answering affirmatively to any of the two first questions is understood as a slight risk of suicide, while answering affirmatively the third question involves a high risk of suicide.

### Procedure

The recruitment of participants was carried out by several University professors and through the main communication channels of the University in order to reach students from different University degrees. No extra credit points or monetary incentives were awarded for participation in the study. The research ethics committee (CER) of the University of the Balearic Islands approved the study and provided full consent. All participants provided explicit consent prior to the beginning of the study.

### Data Analysis

SPSS v23.0 was used for data analysis. For the comparisons regarding the distributions of age groups, gender, ethnicity, degree studies, and yes/no answers for questions about suicide, we report the Fisher's Exact Test (*P)*, a more adequate statistic than χ^2^ when frequency tables contain a considerable number of cells with <5 cases.

After verification of the assumptions, a set of Multiple Analyses of Variance (MANOVAs) with group as the fixed factor (Victim, Aggressor, Control) was carried out for each dependent variable. Power (1-β) is also presented to show the probability of accepting an alternative hypothesis when it is present, which also refers to its sensitivity. Alcohol and cannabis consumption scores were normalized to make both variables comparable, allowing to graphically represent them together in a clearer way.

In order to determine the best predictors of each category (victims, aggressors), a bivariate logistic regression was performed. Odds ratios (OR) and its 95% CI were estimated for each factor.

## Results

### Sociodemographic Data

Regarding sociodemographic characteristics of the sample (see Table 1), the three groups showed distinct frequency distributions according to age (*p* = 0.011) and degree (*p* = 0.001), but not in terms of gender (*p* = 0.147) and ethnicity (*p* = 0.725). *Post-hoc* tests applied to the contingency tables (adjusted standardized residuals with a threshold of *Z* = 1.96, Bonferroni corrected) revealed that there were more past aggressors in the group aged over 25 [*Z* = 2.2; *p* = 0.028] and none in the age range between 24 and 25 years of age [*Z* = −3.1; *p* = 0.002]. The distribution of past victims was disproportionately large in arts and humanities [*Z* = 2.2; *p* = 0.028] and less frequent in social and legal sciences [*Z* = −2.0; *p* = 0.045] as it would be expected by chance. In addition, there were more past aggressors that coursed social and legal sciences [*Z* = 2.2; *p* = 0.028] and less who took health sciences at University [*Z* = −3.1; *p* = 0.002]. Participants in the control group had mainly coursed health sciences [*Z* = 3.1; *p* = 0.002] and showed a disproportionate lower frequency in arts and humanities [*Z* = −2.3; *p* = 0.214] as it would be expected by chance.

### Between-Groups Differences in Measured Variables (G1)

**Figure 1:**
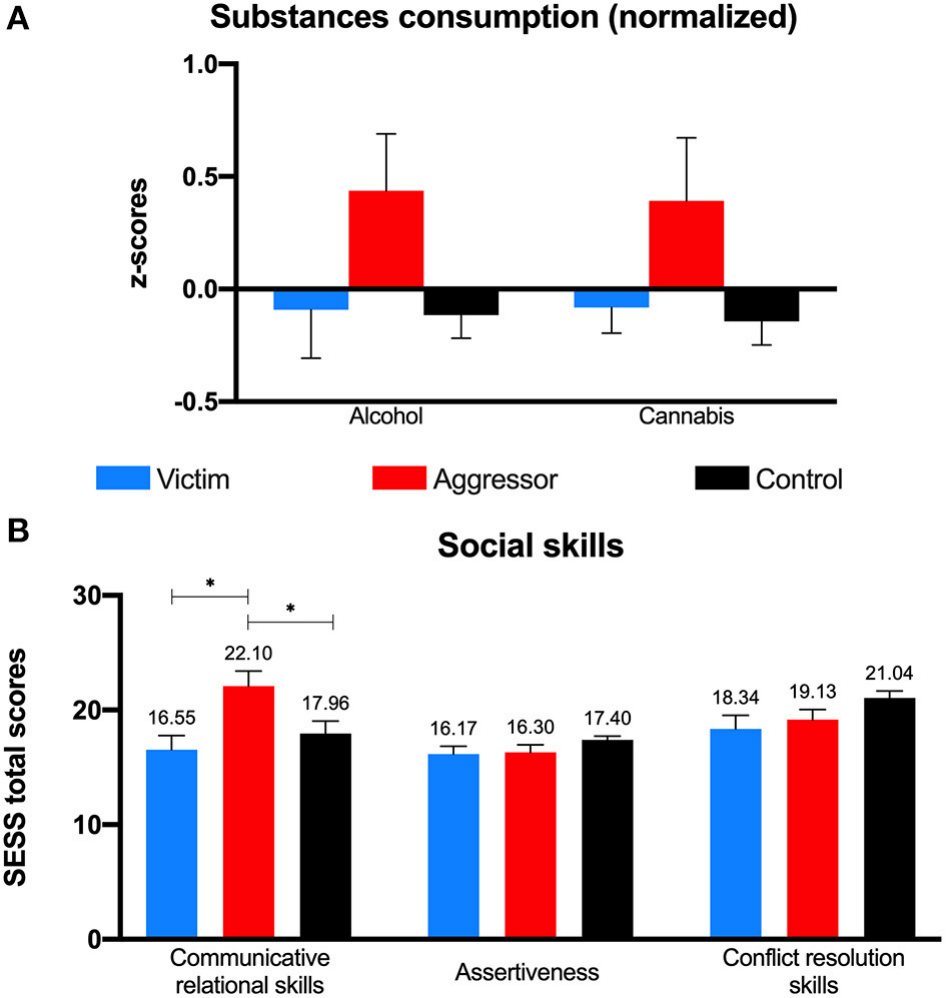
Substance consumption **(A)** and social skills **(B)** by group. Error bars represent Standard Error of Mean; *p < 0.05.

Table 2 shows descriptive statistics for all relevant variables between past victims, past aggressors, and controls (G1). Although aggressors tend to consume more alcohol and cannabis than the other groups, no statistical differences were found between groups regarding substance consumption, neither alcohol nor cannabis (H1); alcohol [*F* = 2.76, *p* = 0.068, $\eta_{\text{p}}^{2}$ = 0.052, 1-β = 0.53], cannabis [*F* = 2.84, *p* = 0.063, $\eta_{\text{p}}^{2}$ = 0.053, 1-β = 0.55] (see Figure 1A).

**Table 2:** Descriptive statistics by group.

**Measure**	**Victim**		**Aggressor**		**Control**	
	***M***	***SD***	***M***	***SD***	***M***	***SD***
Alcohol consumption	3.10	3.96	4.90	4.73	3.02	2.35
Cannabis consumption	0.55	1.53	1.73	3.82	0.40	1.75
Reward sensitivity	8.52	3.43	12.03^**^	3.66	8.42	3.92
Punishment sensitivity	14.62	4.99	11.57	6.43	12	5.62
Emotional sensitivity	15.97	3.08	14.80^*^	4.10	16.76	2.21
Emotional regulation	81.31^**^	20.86	74.17	20.40	66.49	17.05
Emotional lack of control	23.97^**^	10.62	22.13	10.11	17.11	7.95
Emotional rejection	21.72^**^	7.24	18.23	6.93	16.33	5.67
Life interference	14.86	4.01	13.30	5.49	12.91	4.30
Lack of emotional attention	10.48	2.87	10.33	2.28	10.16	2.12
Emotional confusion	10.28	2.83	10.17	1.93	9.98	2.02
Communicative relational skills	16.55	6.60	22.10^**^	7.20	17.96	7.16
Assertiveness	16.17	3.57	16.30	3.70	17.40	2.23
Conflict resolution skills	18.34	6.30	19.13	4.89	21.04	4.07
Psych. adjustment (past)	34.66^**^	11.74	19.70	16.53	-	-
Psych. adjustment (present)	21.79	13.41	19.43	17.76	16.09	14.16
Self-esteem	27.72^*^	6.70	29.43	6.25	31.60	6.60

* *p < 0.05*,

** *p < 0.01*.

For social skills (H2) (Table 2 and Figure 1B), past aggressors reported a higher index of communication and relational skills than both past victims and controls, with a medium effect size [*F* = 5.12, *p* = 0.008, ${\text{η}}_{\text{p}}^{2}$ =0.092, 1-β = 0.81]. Groups did not statistically differ neither in assertiveness [*F* = 1.81, *p* = 0.169, ${\text{η}}_{\text{p}}^{2}$ = 0.035, 1−β = 0.37] nor in conflict resolution skills [*F* = 2.87, *p* = *0.0*61, ${\text{η}}_{\text{p}}^{2}$ = 0.054, 1-β = 0.55].

**Figure 2:**
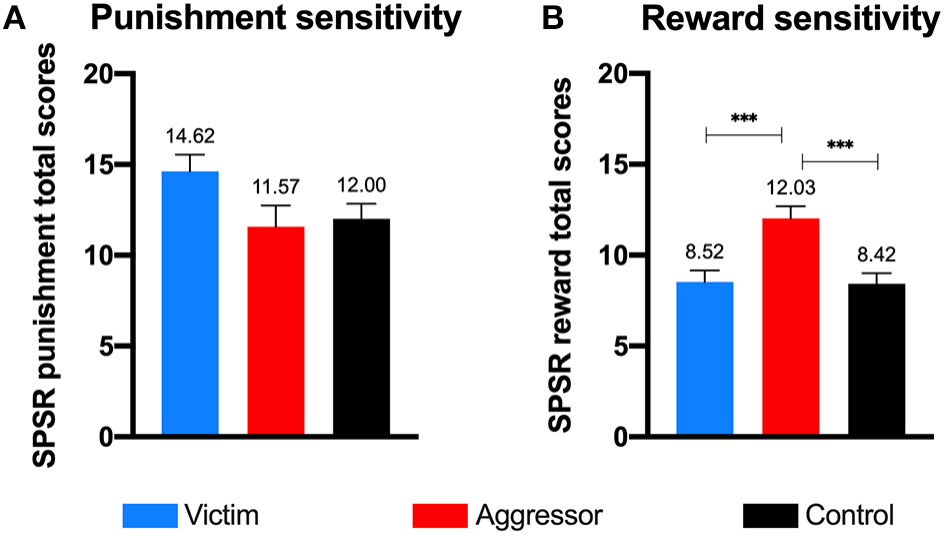
Punishment sensitivity **(A)** and reward sensitivity **(B)** by group. Error bars represent Standard Error of Mean; ***p < 0.001.

Referring to punishment (H3) and reward sensitivity (H4), past aggressors obtained higher scores in reward sensitivity than past victims and controls, with a large effect size [*F* = 9.87, *p* < 0.001, ${\text{η}}_{\text{p}}^{2}$ = 0.164, 1-β = 0.98]. However, the three groups did not significantly differ in terms of punishment sensitivity [*F* = 2.56, *p* = 0.082, ${\text{η}}_{\text{p}}^{2}$ = 0.048, 1-β = 0.50] (see Table 2 and Figure 2).

**Figure 3:**
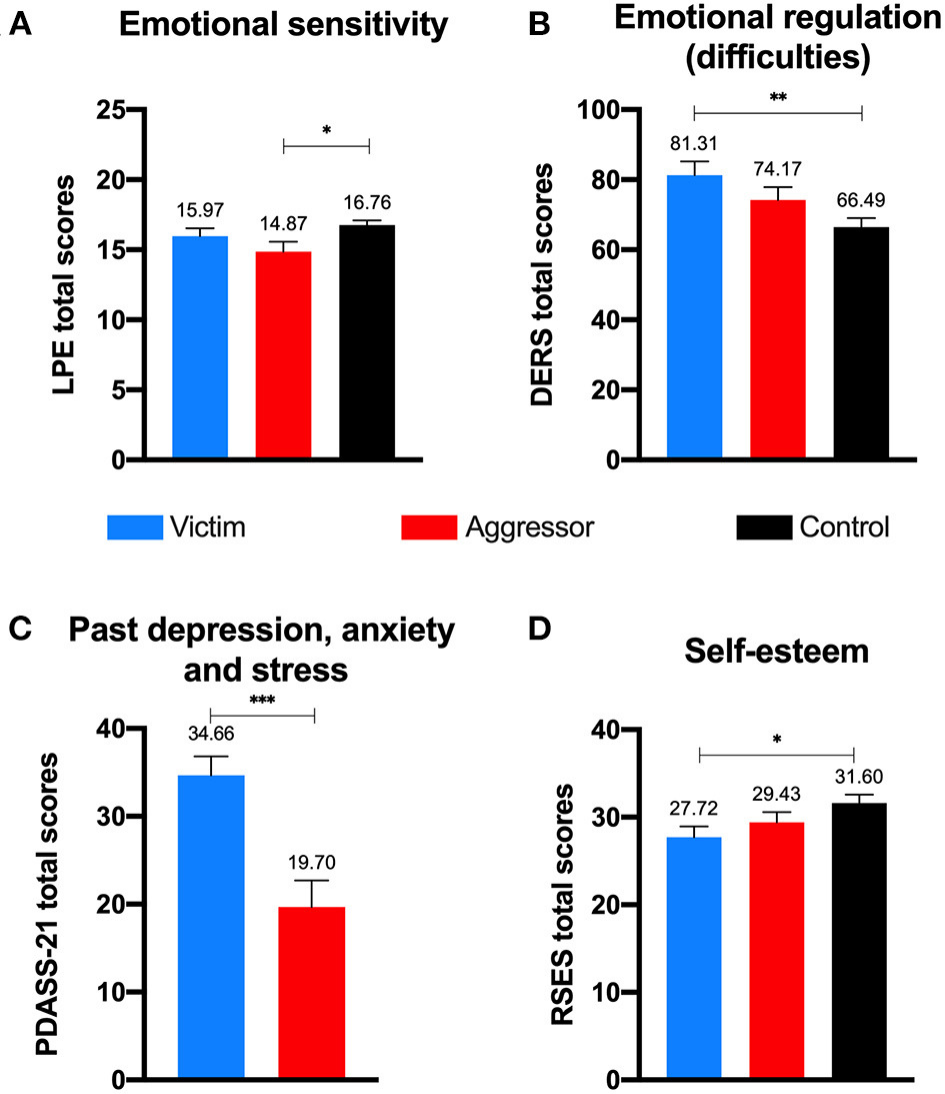
Emotional sensitivity **(A)**, difficulties in emotional regulation **(B)**, psychological adjustment in the past **(C)**, and self-esteem **(D)** by group. Error bars represent Standard Error of Mean; *p < 0.05; **p < 0.01; ***p < 0.001.

For emotion regulation (H5), as measured by the DERS, past victims -but not aggressors- showed more difficulties on emotional regulation than controls (see Figure 3B), with a medium effect size [*F* = 5.38, *p* = 0.006, ${\text{η}}_{\text{p}}^{2}$ = 0.096, 1-β = 0.83]. Breaking down by DERS subscales, results showed that past victims showed larger scores than controls in emotional lack of control [*F* = 5.36, *p* = 0.006, ${\text{η}}_{\text{p}}^{2}$ = 0.096, 1-β = 0.83] and in emotional rejection [*F* = 6.12, *p* = 0.003, ${\text{η}}_{\text{p}}^{2}$ = 0.108, 1-β = *0.8*8], both with medium effect sizes, but not in terms of life interference, lack of emotional attention and emotional confusion (all *p*s > 0.20).

Regarding psychological adjustment (H6) in the past, when the negative situation occurred, measured only in victims and aggressors with the retrospective version of DASS-21, past victims reported to have psychologically suffered more in that moment than past aggressors [*F* = 94.17, *p* < 0.001,${\text{η}}_{\text{p}}^{2}$ = 0.65, 1-β > 0.99], showing a large effect size (Figure 3C). The subscales of the DASS-21(depression, anxiety and stress) revealed larger scores in victims for all variables (all *p*s < 0.002), indicative of problems in anxiety, depression, and stress, also with large effect sizes (0.55, 0.64, and 0.64, respectively). In terms of psychological adjustment in the present moment, groups did not show significant differences neither in the global score [*F* = 1.31, *p* = 0.270, ${\text{η}}_{\text{p}}^{2}$ = 0.025, 1-β = 0.28] nor in any of its subscales (all *p*s > 0.14).

Regarding self-esteem (H7), past victims reported lower scores than controls (but not in comparison to aggressors) with a medium effect size [*F* = 3.21, *p* = 0.044, ${\text{η}}_{\text{p}}^{2}$ = 0.060, 1-β = 0.60], see Figure 3D.

When referring suicidal thoughts (H8) and behaviors in the past, the group of victims scored higher than aggressors and controls to the questions: *Have you ever felt so bad that you wanted to be dead*? (% of yes responses: victims = 75.9%; aggressors = 53.3%; controls = 44.4%), [*P* = 7.21, *p* = 0.028] and *Have you ever tried to hurt yourself?* (% of yes responses: victims = 48.3%; aggressors = 30%; controls = 17.8%), [*P* = 7.65, *p* = 0.024]. The third question “*Have you ever tried to take your own life?”* did not show statistical differences between groups (% of yes responses: victims = 13.8%; aggressors = 10%; controls = 8.9 %), [*P* = 0.59, *p* = 0.852].

Following Table 2 and Figure 3A, past aggressors showed a lower index of emotional sensitivity (H9) as measured with the LPE-Cut in comparison with the control group, but not as compared with past victims, with a medium effect size [*F* = 3.56, *p* = 0.032, η_p_ = 0.066, 1-β = 0.65].

### Main Predictors of Victims and Aggressors (G2)

Finally, to identify the predictive factors for the detection of victim/aggressor categories (G2), all variables showing significant group differences according to the MANOVA were introduced in a logistic regression analysis, being the dichotomic dependent variable the category “victim” *v*s. “aggressor.” The model was significant [χ^2^ (6) = 31.12, *p* = 0.003, *R*^2^ Nagelkerke = 0.55] and classified correctly 81.4% of cases (89.7% of victims and 73.3% of aggressors). The model revealed that higher sensitivity to reward [*OR* = 1.39, 95% *CI* 1.12–1.72; *p* = 0.003], was associated with being classified as an aggressor. This means that adding 1 point to this scale was associated with an increase of 39% in being identified as an aggressor. Similarly, the total measure of depression, anxiety and stress in the past was associated with being classified as a victim [*OR* = 0.91, 95% *CI* 0.85–0.98, *p* = 0.012]. This means that subtracting 1 point to this scale was associated with a decrease of 9% in being identified as a victim. In sum, the variables that significantly predicted the classification as either an aggressor or a victim were sensitivity to reward and low psychological adjustment in the past, respectively.

## Discussion

The main goals of this work were to retrospectively analyze the putative effects of having suffered or perpetrated bullying in the past in a sample of young adults (G1) and to identify the best predictors of being classified as a victim or as an aggressor (G2). For this purpose, several individual factors were selected to explore whether young people and adults continue to present the related bullying and victimization profiles in the medium or long-term, in contrast to a control group.

For our first goal (G1), in a general way, we claim that adults who bullied or were bullied in the past tend to report similar long-term outcomes than those reported in the literature for children and adolescents at the short-term and those shown in longitudinal studies. Particularly, although past victims and past aggressors do not show a significantly higher alcohol and cannabis consumption than controls, past aggressors show a tendency to higher substance consumption, as we hypothesized (H1). Regarding social skills (H2), contrary to our expectations, declared aggressors obtain better scores in relational skills. Also, former aggressors show larger sensitivity to reward (H4) and emotional insensitivity, as expected (H9). Meanwhile, victims show larger sensitivity to punishment (H3), a poorer emotional regulation (H5), lower psychological adjustment in the past (H6), lower self-esteem (H7) and higher frequency of suicidal thoughts (H8). Next, we are going to consider and discuss the relevant variables individually.

First, and contrary to our expectations and the extant literature, former victims of bullying did not present larger scores in substance use in adulthood (H1). Regarding past aggressors, they present a tendency, although not significant, to higher consumption of alcohol and cannabis than controls, according to the literature (Sigurdson et al., 2014; Antoniadou et al., 2016). A possible explanation for this outcome is that substance consumption is higher in young populations and decreases during adulthood (Ministerio de Sanidad Consumo y Bienestar Social, 2018).

We also predicted that social skills (H2) would be poorer in both past aggressors and victims of bullying, as reported in some works (Sandoval et al., 2015; Kljakovic and Hunt, 2016). In this case, results did not confirm our hypothesis, which could indicate that adults who have suffered bullying in the past can strive to slightly improve their repertoire of social interaction abilities in case these were poor. On the other hand, and contrary to our expectations, aggressors showed even larger communication and relational skills than controls. A possible explanation for this result is that aggressors, who tend to score higher in both extraversion and sensation seeking (van Geel et al., 2017a; Dåderman and Ragnestål-Impola, 2019) perceive themselves as more socially skilled. Moreover, some authors (Sutton et al., 2001), state that bullies need high social cognition and skills for understanding others, in order to manipulate and direct other people, while avoiding to be punished by their aggressive behavior, not presenting a social deficit. In this vein, the theory of nasty minds (Happé and Frith, 1996; Lonigro et al., 2014; Wang and Wang, 2015) state that children with behavior problems can show an altered social insight in social situations, which leads them to low emotional sensitivity and to excel in some antisocial behaviors (such as lying or bullying) that can be useful in some social contexts. Our data concord with this theory, as aggressors seem socially skilled for managing social contexts, despite using antisocial behaviors. Also, they could have a good social perception of themselves, a finding aligned with Sutton et al. (2001) alternative view. Future works might want to better elucidate this controversial result by including the measurement of social skills with objective measures, rather than self-reports.

We also expected that former victims would present a larger sensitivity to punishment than non-victims (H3). However, our results do not endorse this statement. A possible explanation to this result could be that a long-term avoided punishment has led, to some extent, to normalize the baseline levels of sensitivity, or that a long-term habituation has occurred, derived from the impossibility to escape from aversive situations. Future studies should address this question. Complementarily, we hypothesized that past aggressors would present a larger sensitivity to reward (H4), following previous reports (Antoniadou et al., 2016; Poon, 2016). Our results supported this hypothesis with a large effect size. In fact, sensitivity to reward was the most important variable to classify participants as aggressors, following the logistic regression analysis. This is an expected result, as bullies are more prone to be involved in risky behaviors and might be more eager to sensation seeking (Antoniadou et al., 2016).

With respect to emotional regulation (H5), past victims presented a higher rate of emotionally maladaptive strategies in the face of conflicts, consistent with our predictions. Difficulties with emotion regulation in victims are specifically relevant in terms of a more pronounced emotional lack of control and in a more prominent rejection of emotions. These difficulties in emotional regulation could be interpreted as a previously low perceived emotional consciousness that increases the risk of being involved in bullying situations, as alluded by Elipe et al. (2012). Alternatively, other explanations take into account that social support might facilitate coping and recovery from traumatic events (Ystgaard et al., 1999). Thus, it is likely that the loss of such social support experienced by victims, manifested by exclusion in bullying situations, may represent a traumatic event with long-term emotional repercussions (Lev-Wiesel et al., 2006), such as the higher emotional lack of control and emotional rejection found in former victims of bullying. Finally, other theoretical approaches, such as the Social Information Processing (SIP) model by Crick and Dodge (1994), might provide additional clues for the self-reported difficulties in emotional control in victims of bullying. After the encoding of the social situation and the interpretation of social cues has taken place (steps 1 and 2), the clarification of goals is defined (step 3), and act as a focused arousal state to guide behavior. In this sense, former victims of bullying might experience difficulties in controlling their aroused emotional states that might lead to a maladaptive response strategy.

We also predicted that participants with a history of victimization would present higher scores on depression, anxiety and stress than controls (H6). This prediction was confirmed, but only when referring to the past, when participants were suffering victimization associated to bullying. It is also worthy of note that the global measure of depression, anxiety and stress (in the past) was the most relevant variable in the logistic regression analysis that allowed for the correct classification as a victim of bullying. In this vein, these results are compatible with extant literature (Forster et al., 2013; Stuart and Jose, 2014; Sandoval et al., 2015) as being bullied has a relevant influence on the onset of depression, anxiety and stress. Nevertheless, some authors (for a review, see Rivara and Le Menestrel, 2016) defend another pathway focused on a symptoms-first approach: children and adolescents who firstly present depression and anxiety symptoms are more prone to suffer bullying. This pathway focused on vulnerability is compatible with our results, as these variables are adequate to classify participants as victims, following the logistic regression analysis.

In terms of self-esteem (H7), the present results mirror previous studies (Tsaousis, 2016) showing that former victims of bullying present a current lower self-esteem than controls. In this vein, a meta-analysis (Tsaousis, 2016), pointed that the relationship between bullying and self-esteem is stronger in adolescence than during childhood. The present results suggest that lower self-esteem in former victims of bullying also remains in adulthood.

As expected, former victims of bullying presented a larger rate of suicidal thoughts and willingness to hurt themselves in the past than aggressors and controls (H8). However, this was not the case for the risk of committing suicide, although it correlates with being currently bullied (Shin Kim and Leventhal, 2008). Thus, the current risk of suicide seems not to be related to having been bullied it in the past, at least without the presence of psychiatric problems (Alavi et al., 2015).

Finally, referring to antisocial traits (H9), results confirm that past aggressors present a lower index of emotional sensitivity than controls, a nuclear characteristic in this type of phenomena (Antoniadou et al., 2016; Garaigordobil, 2017). As previously stated, the theory of nasty minds and its relationship with antisocial behaviors can help to gain insight into the relationship between low emotional sensitivity and bullying, also in the long-term (Happé and Frith, 1996). Antisocial behaviors and low emotional sensitivity can be a sign of “nasty minded” people, in opposition to “nice minded” people, who better characterized for morality and emotional sensitivity (Wang and Wang, 2015). “Nasty minded” people would present the callous/unemotional trait not only during childhood, but also in adolescence and even adulthood. Moreover, prosocial behaviors and the understanding mental states of others can come to a halt and even decrease in adulthood (Bernstein et al., 2011; Shakoor et al., 2012; Hao and Liu, 2016; van Geel et al., 2017b).

Regarding the second main objective of this study, related to the predictors of being classified as a victim or as an aggressor (G2), we report one key variable for each group, according to the logistic regression. On the one hand, the total score on depression, anxiety and stress in the past was the most prominent variable to be classified as a victim. On the other hand, reward sensitivity was the best predictor of being identified as an aggressor. These findings remark the importance of improving psychological well-being and address reward sensitivity or sensation seeking during childhood and adolescence, in order to prevent and even intervene in bullying situations.

As a secondary finding, our results show that the prevalence of childhood victimization associated to bullying obtained retrospectively (20%) is comparable to that reported in large-scale and meta-analytic studies, which estimate that about 30% of children report to have been bullied in the previous months (Craig et al., 2009; Biswas et al., 2020).

Despite these interesting findings, the present work has several limitations. The first refers to the moderate sample size, as some of the effect sizes obtained were moderate. Second, it is possible that social desirability and retrospective bias might have influenced participants' responses, especially when it comes to delicate topics, such as bullying and aggression, as measured with self-reports. Also, further works might want to explore victimization in the workplace and to ask participants whether they had received psychological and/or psychiatric treatment because of bullying victimization. Finally, it would be adequate to consider the mixed “bully-victim” profile in future studies. Adding this information could help to better comprehend the impact of long-term bullying and victimization, and to deal better with this social scourge in the short and the long term.

## Conclusions

In conclusion, the main findings for this study show that variables related to bullying are not only present when the phenomenon occurs, but also when those who suffer or perpetrate it grow up. On the one hand, former victims of bullying present higher difficulties in emotional regulation and lower self-esteem than controls and former aggressors. On the other hand, former aggressors present higher emotional insensitivity and sensitivity to reward, which is the main predictor of being a classified as an aggressor. Considering these results, and being bullying a widespread and worrying phenomenon, the present results could be useful for both its understanding and prevention.

According to our results, we advise to provide children and adolescents with adaptive emotional strategies in front of conflicts and methods to increase psychological well-being and self-esteem, such as cognitive reappraisal, identifying context cues, and clues to reduce physiological responses, such as deep breathing and full attention (Aldao, 2013; Gross, 2015; Pascual Jimeno and Conejero López, 2019). Training in emotional regulation and cognitive appraisal is also advisable for aggressors, in order to diminish the need for sensation seeking and to reduce emotional insensitivity (Borjali et al., 2016). Moreover, the stimulation of empathy as a measure to address antisocial traits in youngsters, can be also useful in bullying intervention (van Geel et al., 2017b). Finally, the early detection of suicidal thoughts will be of crucial importance to prevent severe possible outcomes of bullying situations.

## Data Availability Statement

The raw data supporting the conclusions of this article will be made available by the authors upon request, without undue reservation.

## Ethics Statement

This study was approved by the Comitè de Recerca de les Illes Balears (CER). All participants provided their explicit consent and volunteered to participate in the present study.

## Author Contributions

MV-P designed the study, collected and curated the data, and wrote the first draft of the manuscript. AF analyzed the data, wrote, and corrected the manuscript. MS designed the study and corrected the manuscript. EA-M wrote and corrected the manuscript. DA-R designed the study, analyzed the data, wrote and corrected the manuscript. All authors contributed to the article and approved the submitted version.

## Conflict of Interest

The authors declare that the research was conducted in the absence of any commercial or financial relationships that could be construed as a potential conflict of interest.
